# Next-Generation Sequencing at High Sequencing Depth as a Tool to Study the Evolution of Metastasis Driven by Genetic Change Events of Lung Squamous Cell Carcinoma

**DOI:** 10.3389/fonc.2020.01215

**Published:** 2020-08-05

**Authors:** Hicham Mansour, Abdelhak Ouhajjou, Vladimir B. Bajic, Roberto Incitti

**Affiliations:** ^1^GES-LCM2E, FPN, Mohamed First University, Oujda, Morocco; ^2^Al-Azhar Oncology Center, Rabat, Morocco; ^3^CBRC, King Abdullah University of Science and Technology (KAUST), Thuwal, Saudi Arabia

**Keywords:** lung cancer, NGS, FGFR, HRAS, CDKN2, tumor evolution

## Abstract

**Background:** The aim of this study is to report tumoral genetic mutations observed at high sequencing depth in a lung squamous cell carcinoma (SqCC) sample. We describe the findings and differences in genetic mutations that were studied by deep next-generation sequencing methods on the primary tumor and liver metastasis samples. In this report, we also discuss how these differences may be involved in determining the tumor progression leading to the metastasis stage.

**Methods:** We followed one lung SqCC patient who underwent FDG-PET scan imaging, before and after three months of treatment. We sequenced 26 well-known cancer-related genes, at an average of ~6,000 × sequencing coverage, in two spatially distinct regions, one from a primary lung tumor metastasis and the other from a distal liver metastasis, which was present before the treatment.

**Results:** A total of 3,922,196 read pairs were obtained across all two samples' sequenced locations. Merged mapped reads showed several variants, from which we selected 36 with high confidence call. While we found 83% of genetic concordance between the distal metastasis and primary tumor, six variants presented substantial discordance. In the liver metastasis sample, we observed three *de novo* genetic changes, two on the FGFR3 gene and one on the CDKN2A gene, and the frequency of one variant found on the FGFR2 gene has been increased. Two genetic variants in the HRAS gene, which were present initially in the primary tumor, have been completely lost in the liver tumor. The discordant variants have coding consequences as follows: FGFR3 (c.746C>G, p. Ser249Cys), CDKN2A (c.47_50delTGGC, p. Leu16Profs^*^9), and HRAS (c.182A>C, p. Gln61Pro). The pathogenicity prediction scores for the acquired variants, assessed using several databases, reported these variants as pathogenic, with a gain of function for FGFR3 and a loss of function for CDKN2A. The patient follow-up using imaging with 18F-FDG PET/CT before and after four cycles of treatment shows discordant tumor progression in metastatic liver compared to primary lung tumor.

**Conclusions:** Our results report the occurrence of several genetic changes between primary tumor and distant liver metastasis in lung SqCC, among which non-silent mutations may be associated with tumor evolution during metastasis.

## Background

Lung cancer is the most common type of cancer and the leading cause of cancer-related deaths for both men and women in the world (1, 2). There are two major types of lung cancer, namely, non-small cell lung carcinoma (NSCLC) and small cell lung carcinoma. NSCLC can be further divided into three main subtypes: large cell lung carcinoma, squamous cell carcinoma, and adenocarcinoma. Lung SqCC accounts for almost 30% of NSCLC, while there are also several other types that occur less frequently (3, 4). Significant progress on cancer research was made possible by the development of next-generation sequencing (NGS) (5–8) and, subsequently, by extensive research efforts on several tumors and patients (9–11). The body of knowledge on cancer genetics has increased sizably, and, depending on cancer type, researchers have identified several target genes that could be involved in cancer development, to improve patient care and to enhance the survival rate (12–16). Clinical research efforts to provide a comprehensive landscape of molecular alterations of lung SqCC have successfully characterized this cancer at the genetic level (9, 17). Researchers from the Cancer Genome Atlas (TCGA) identified several genes that are aberrantly expressed and/or mutated in lung SqCC patients. Major alterations have been described in TP53, PI3K, CDKN2A, SOX2, CCND1, ERBB, MET, DDR2, and FGFR1 (18–29). In particular, identification of genetic amplification in FGFR1, mutations in ERBB family, and abnormalities in PI3K have raised considerable interest in view of applications to the therapeutic management of SqCC patients. Furthermore, in multiple clinical trials, some of these genetic alterations have been targeted and have shown promising progress in treating SqCC (LUME-Lung 1, LUX-Lung 8, BASALT-1 phase II, FLEX, SQUIRE, and others) (30–36). Unfortunately, the observed benefits have been sporadic and heterogeneous, leading to some disappointment (18). Recently, the understanding of the complex interactions between the immune system and cancer has led to the development of immune checkpoint inhibitors (ICI). The combination of chemotherapy and ICI, in the first-line treatment of metastatic SqCC, has shown higher overall survival (OS) and progression-free survival (PFS) than the use of chemotherapy alone (37–39). Unfortunately, several studies have shown that only a subset of patients could benefit from this therapeutic protocol, such as patients with wild-type EGFR or negative ALK rearrangements (40, 41). Moreover, in different PD-L1 expression subgroups, the benefit in OS and PFS is mixed (42).

Because of the heterogeneity of the tumor at genetic and molecular levels, treatment of SqCC continues to remain a challenge. There may be several reasons due to the existence of different genomic patterns in SqCC, and the genetic targets that have been found so far are probably not driver mutations and are therefore currently unactionable or “undruggable” mutations for this disease (18). Several available treatments do not exhibit efficacy in metastasis lung SqCC, leaving these patients with fewer and inefficient treatment options, a situation, which could explain the poor survival and the frequent relapse of these patients. Thus, the identification of potential targets in SqCC has become urgent and essential.

The spread of cancer cells from primary tumors to other vital organs is often associated with poor prognosis (43, 44). Given its potential impact on patient care, understanding the genetic differences between primary tumors and distant metastasis is critical and will have value in terms of treatment strategies. A better understanding of the dynamic of the molecular evolutionary process of the lung SqCC will provide greater insight toward the development of a suitable therapeutic guide (45, 46). In this study, we used tumor genetic data generated by NGS, to investigate if tumor heterogeneity occurs between primary tumor and liver metastatic sites in lung SqCC and to detect non-silent mutations, which may be associated with tumor evolution, and its evolutionary trajectory from primary tumor through relapsed disease.

## Case Presentation

### Patient

A 72-year-old African man was presented to our clinic in 2016, with a worsening dyspnea and chronic dry cough. Symptoms included fatigue and muscle atrophy, with pain in the chest and right rib cage. He suffered from cervical disc herniation and frequent reversible heart palpitation. The patient was a former smoker, consuming one pack per day for 16 years until he was 32 years old. In the previous two years, he had a significant weight loss of 20 kg. The physical exam shows a decrease in vesicular murmur and vocal vibrations of the right lung with no evidence of lymphadenopathy. The abdominal echography shows hepatomegaly. A suspicious mass for pulmonary neoplasm was found on chest X-ray. A CT scan detected a liver mass of 6 cm in the segment IV. Two biopsies were performed, one in the mediastinum (sample 69380-1-S7) and another in the liver (sample 69381-2-S8), and pathological examination confirmed the presence of squamous cell lung carcinoma, NSCLC at stage IVB (T4N2aM1c). The patient underwent FDG-PET scan imaging as part of the routine diagnosis and staging, before starting the chemotherapy treatment. Two hypermetabolic masses were localized; the maximum standardized uptake value (SUVmax) was assessed for these masses. On 26th December 2016, he received the first cure with paclitaxel–carboplatin. On 31st December 2016, he was admitted in the hospital for dysenteric syndrome and hemorrhoids and was treated with loperamide, diosmectite, metronidazole, lidocaine hydrochloride, diosmin, and hesperidin. On 19th January and 9th February 2017, he received the second and third cure with paclitaxel and carboplatin, respectively. Subsequently, on 13th March 2017, a second FDG-PET scan was performed to assess tumor progression. On 16th March 2017, he received the fourth cure with paclitaxel–carboplatin. A complete blood count was performed before each treatment, which was normal. A poor tolerance to chemotherapy was observed, as characterized with the following: nausea, diarrhea, loss of appetite, overall condition deterioration, and neurological disorders. Thus, the combination of immune checkpoint inhibitor and chemotherapy was not recommended and PD-L1 expression level was not investigated. Tyrosine kinase inhibitors (TKIs) were suggested instead. The patient refused to continue with the treatment. Nevertheless, he gave his consent to conducting the genetic analysis and for the publication of the present case report.

### Sample Processing

Microdissections from formalin-fixed paraffin-embedded (FFPE) specimens of these biopsies were performed, ensuring that a tissue area that contained a high percentage of tumor cells would be used for DNA extraction. gDNA was extracted using the QIAamp DNA Micro Kit (Qiagen), then the sequencing library was prepared and was submitted for sequencing on MiSeq (Illumina). We used an existing commercial gene panel test, the somatic Tumor Hotspot MASTR™ Plus with MID for sequencing library preparation (Multiplicom/Agilent). The assay developed for the identification of SNVs in 26 frequently mutated genes in solid tumors. This NGS assay was designed with the input from the French INCA centers (https://www.agilent.com/cs/library/datasheets/public/TUMOR%20HOTSPOT%20MASTR%20PLUS%205991-8378ENN.pdf). This panel targets the following 26 genes: ALK, PTEN, PIK3R1, EGFR, NRAS, STK11, ERBB4, ERBB2, AKT1, HIST1H3B, HRAS, PDGFRA, MAP2K1, CDKN2A, IDH1, H3F3A, IDH2, BRAF, PIK3CA, KIT, CTNNB1, KRAS, FGFR3, MET, FGFR2, and DDR2.

### Data Analysis

We filtered the sequencing reads with a Q-score higher than 20, merged the read pairs, and mapped them on the human genome reference GRCh37/hg19. We filtered out duplicate reads, resulting from clonal amplification of the same fragments during library construction, and the regions which showed mapping coverage of less than 50×. We performed variant detection analysis for SNPs, insertions, and deletions using Sophia Genetics DDM platform v4.5. Only high-confidence variants were retained according to the following criteria: (1) occurring in a high-complexity region, (2) having high, or higher than expected, variant fraction (germline), (3) having a sequencing coverage >50 ×, (4) having >50 supporting reads, and (5) being aligned inside the target region. The copy number variation analysis was not investigated in the present study. Variants were ranked by concordance and discordance between the two samples, and annotation information such as coding consequences was taken forward for further analysis. For each variant, we also reported the variant frequency, defined as variation percent = (altNum/depth) × 100, where “altNum” is the number of reads containing the variant of interest and “depth” is the sum of number of reads containing the variant and the reads covering the reference allele. To obtain pathogenicity prediction scores, the target variants were submitted to the following databases made available by the Sophia Genetics DDM platform: COSMIC, Clinvar, OncoKB, cBioPortal, Varsome, Mutationassessor, Provean, FATHMM, MutationTaster, FATHMM-MK, MetaSVM, MetalR, LRT, SIFT, dbSNP, ClinVitae, and Provean. The protein structures encoded by each mutated gene were submitted to the SMART tool (47), to describe the biological consequence of each mutation on the functional domain of the protein.

## Results

### Image Analysis With 18F-FDG PET/CT

**Figure 1:**
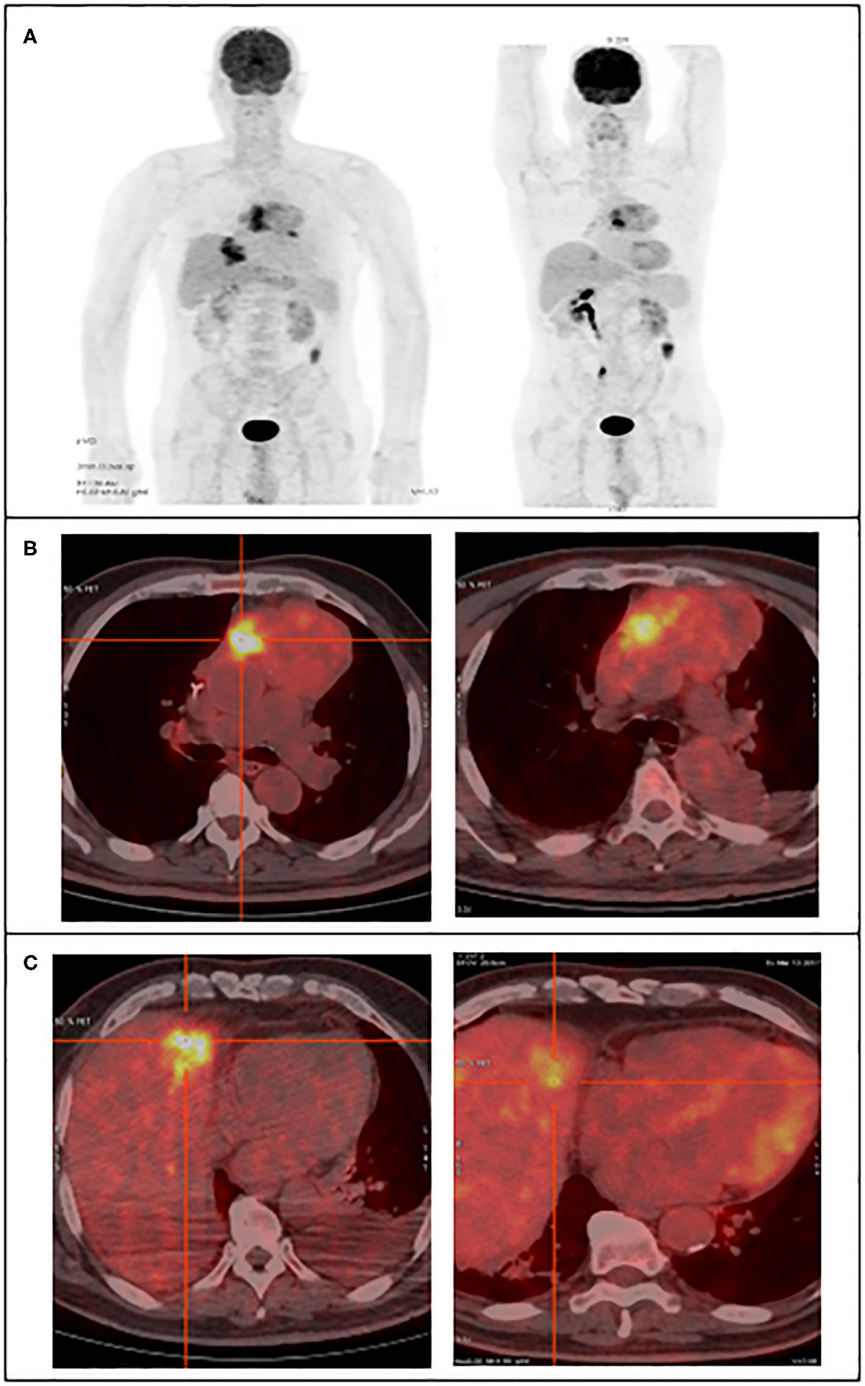
18F-FDG PET/CT images reveal the large hypermetabolic masses, in the superior mediastinum and the hepatic parenchyma, left figures before treatment and right figures post-treatment. **(A)** Maximum intensity projection image, **(B)** transaxial fusion image showing the mediastinal tumor, **(C)** transaxial fusion image showing the liver tumor.

Two intense hypermetabolic masses were revealed by 18F-FDG PET/CT (Figure 1). They were found into the antero-superior mediastinum and in the hepatic parenchyma. The SUVmax of the hypermetabolic masses was assessed before and after treatment. Before treatment, the SUVmax of the mediastinal mass was 7.3 and the hepatic mass was 9.4. After treatment, the mediastinal mass increased to 10.4, while the liver hypermetabolic mass decreased significantly to 3.9. While the primary lesion showed continuous tumor progression, the liver lesion showed significant improvement.

### Sequencing Data Statistics

**Figure 2:**
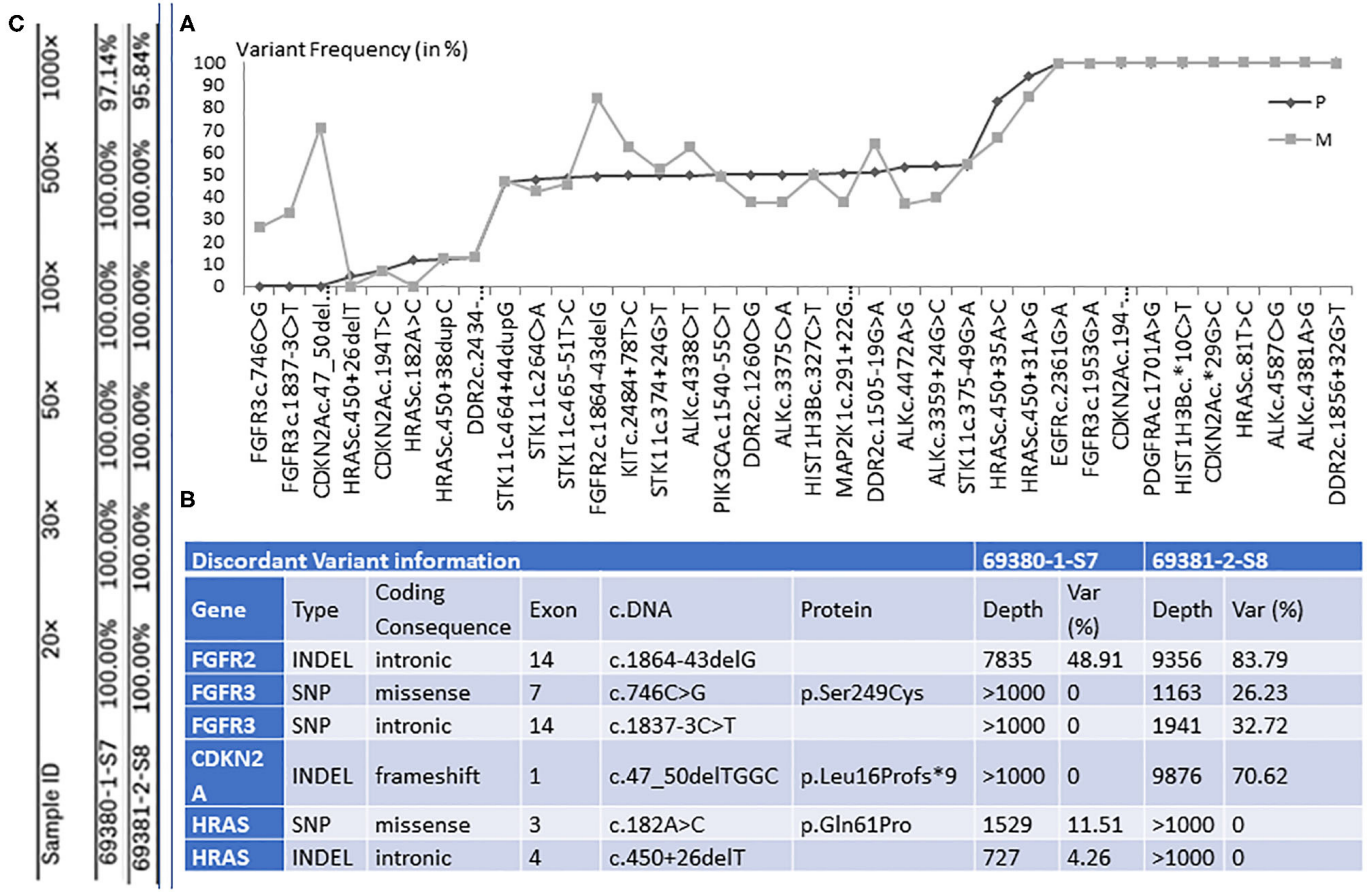
**(A)** The graphical representation of retained genetic variants and their frequency (var %), in primary tumor (P) and distal metastasis (M). **(B)** The illustration of the six discordant variants, among which three with coding consequences. Table **(C)** shows the percentage of target regions with certain coverage for both samples.

The mediastinal and liver samples were sequenced, resulting in 1,661,981 and 2,260,216 read pairs, respectively. Ninety percent of the total reads were above Q35, which is considered as a high threshold for read quality. The mapping of merged reads on the targets genes resulted in 1,628,242 reads (>97%) successfully mapped for the mediastinal sample and 2,134,774 reads (>94%) for the liver sample. In the present experiments, we achieved a high sequencing coverage of the target region. The mean target coverage in this study was around 6,000×. The target region coverage distribution for both samples is shown in Figure 2C and Figure S1.

### Finding Common and Discordant Variants

In the lung sample, 12 out of the 26 genes sequenced presented with genetic changes, with 33 variants retained for high-confidence call and 28 discarded. In the liver sample, 13 genes presented with genetic changes, with 36 variants found and 24 discarded. The comparison between the distal metastasis and the primary tumor shows 30 shared variants and 6 distinct variants. When analyzing the variant frequency of the shared variants and using primary tumor data as baseline, we can observe several clusters (Figure 2).

**Figure 3:**
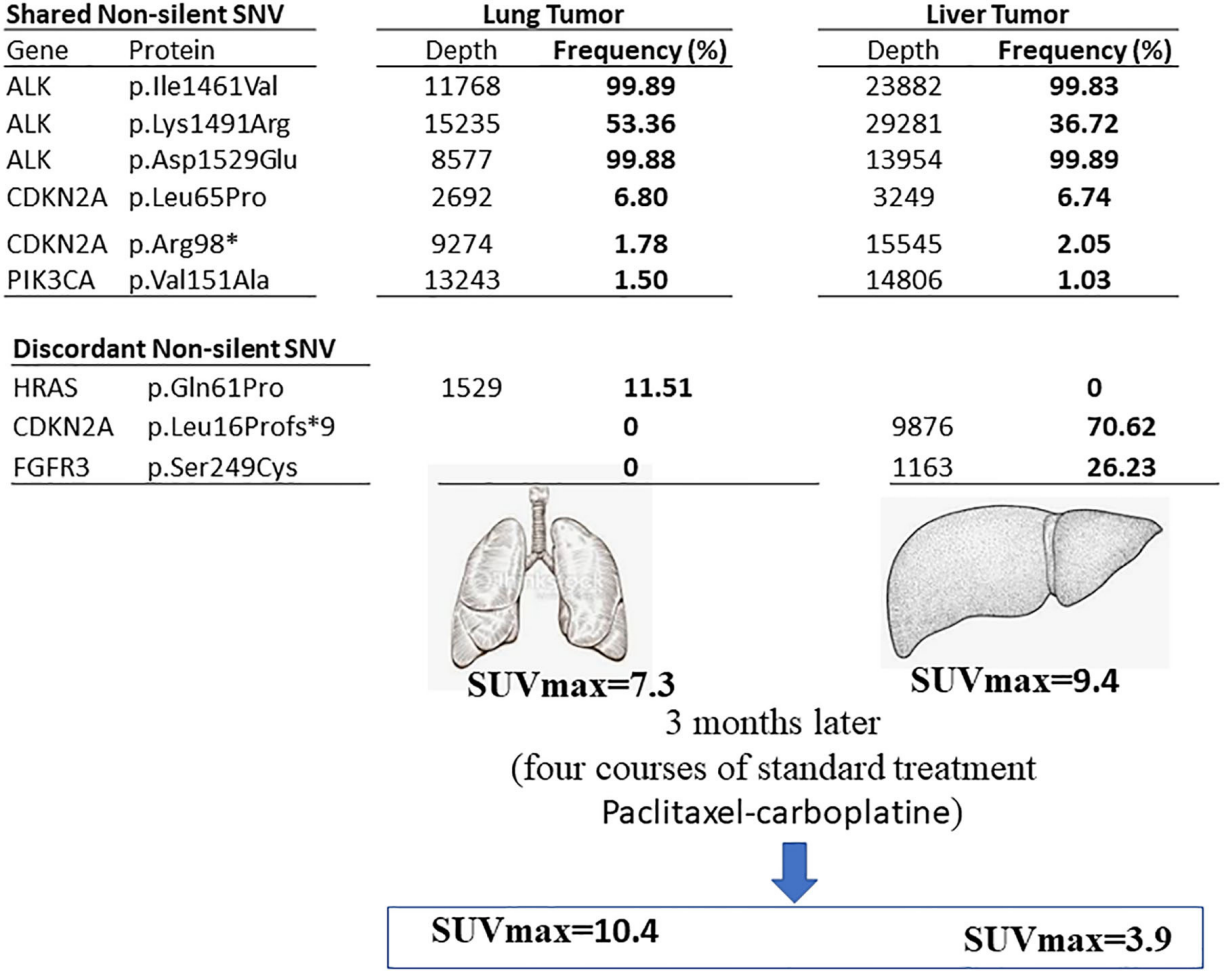
Summary of the current finding. The genetic variants found in primary lung tumor (left) and liver (right) metastasis sites in the lung SqCC patient. The shared and discordant non-silent SNV are mentioned with the frequency percentage.

We found 30 variants shared between primary tumor and metastasis, distributed across 12 genes, and having a similar frequency between the two samples. Among these variants, 6 have genetic changes in the coding sequence of the following genes: ALK (3 variants), CDKN2A (2), and PIK3CA (1) (Figures 2, 3).

We found six discordant variants, covering the following genes: FGFR2, FGFR3, CDKN2A, and HRAS. Among these variants, only three variants had coding consequences as follows: a missense c.182A>C (p.Gln61Pro) on the HRAS gene, a frameshift c.47_50delTGGC (p.Leu16Profs^*^9) on CDKN2A gene, and a missense c.746C>G (p.Ser249Cys) on the FGFR3 gene. The HRAS (p.Gln61Pro) variant is found in primary lung tumor with a frequency of 11.5% and completely absent in the liver metastasis. The CDKN2A (p.Leu16Profs^*^9) and FGFR3 (p.Ser249Cys) variants are present only in the liver metastasis at a frequency of 70.6 and 26.2%, respectively (Figures 2, 3). The full list of variants is publicly available in Clinvar database (www.ncbi.nlm.nih.gov/clinvar/), under the accession numbers SCV001250917 to SCV001250974.

### Pathogenicity Prediction

**Figure 4:**
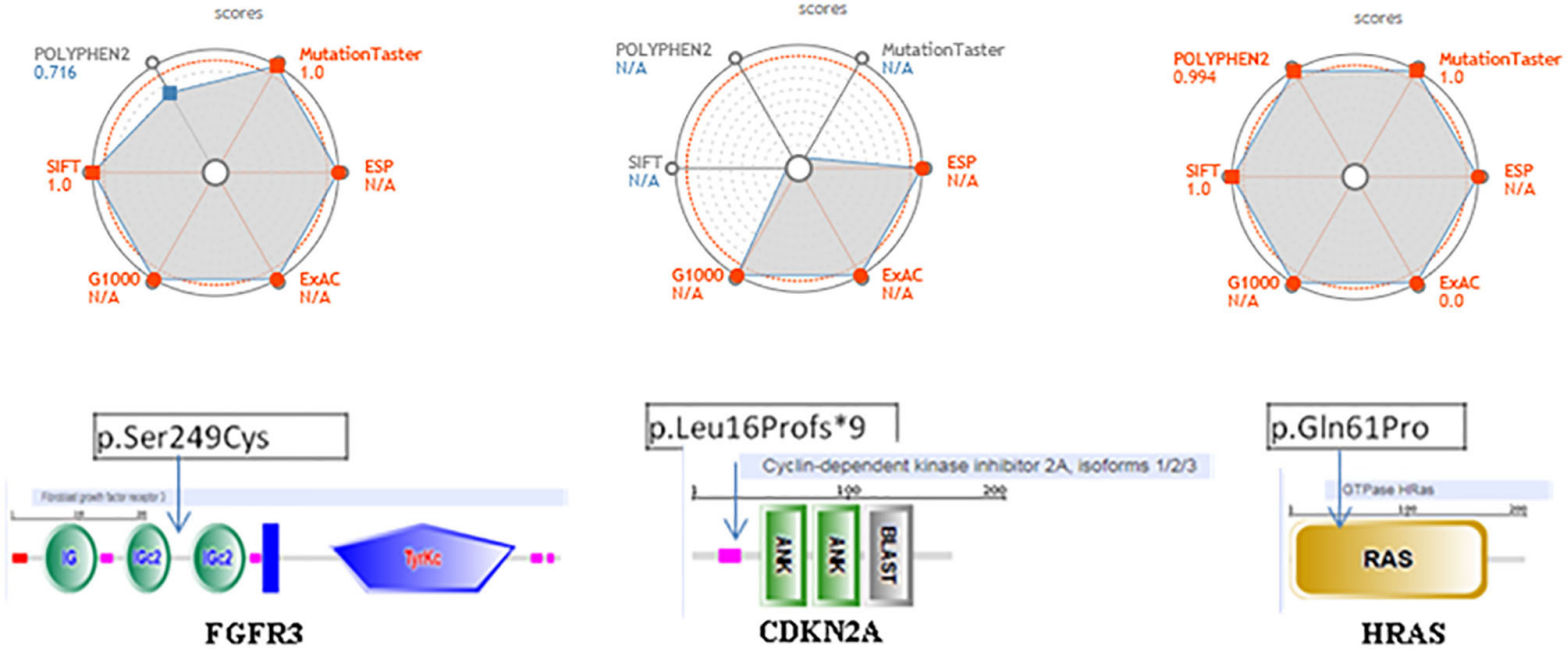
Pathogenicity web plotting prediction scores for the three variants, FGFR3 (p.Ser249Cys) (left side), CDKN2A (p.Leu16Profs *9) (middle side), and HRAS (p.Gh161Pro) (right side). Values are scaled so that the most pathogenic scores are plotted toward the external circles. For POLYPHEN2, Mutation Taster and SIFT, those values approach 1; whereas for ESP, G1000, ExAC they approach. The greater the gray area, the variant is more likely pathogenic. ESP, G1000, ExAC empty values are considered as NIA. Score > 0.9 and population databases (if applicable) <0.1 are shown in red. In the bottom, we show the localization on these three of genetic changes events FGFR3, CDKN2A, and HRAS using SMART database.

The pathogenicity prediction of the three discordant variants with coding consequences shows the following:

1/ The FGFR3 missense c.746C>G (p.Ser249Cys) is located between two functional domains, in a relatively conserved position, and is predicted to have a damaging, disease-causing, and high effect on protein function (Figure 4). This is the most frequently observed FGFR3 variant found in cancer studies and is reported as oncogenic with a gain of function. The pathogenicity prediction of this genetic variation confirmed its association with several cancers, including tumors of the lung and other tumors: the urinary bladder, the urinary tract, the head, the neck, the upper aero-digestive tract, the thymus, and the cervix. This genetic variant is reported as a recurrent hotspot mutation in these tumors. It could be either germline or somatic and is classified as a pathogenic allele.
2/ The CDKN2A, frameshift c.47_50delTGGC (p. Leu16Profs^*^9), is located at a relatively conserved position and predicted to have a high effect on protein function (Figure 4). The variant has a damaging, disease-causing effect on protein function and has been shown to be involved in pancreas carcinoma and thymoma. It has been reported as both somatic and germline mutation and has been classified as a pathogenic allele, associated with hereditary cancer predisposing syndrome.
3/ The variant HRAS, missense on c.182A>C (p. Gln61Pro), is located in the Ras domain and has been found to be expressed in the upper aero-digestive tract and in thyroid cancers (Figure 4). This variant is reported in both germline and somatic and is classified as a pathogenic allele. It has a damaging, disease-causing effect on protein function. We did not find relevant hits for this particular variant in OncoKB, cBioPortal, or My Cancer Genome.

## Discussion

Extensive clinical research efforts and advancements that have been made since the human genome was sequenced have greatly increased our knowledge on cancer genetics. Several genetic alterations have been shown to initiate and sustain specific deregulated cellular signaling pathways involved in various tumors. In the present study, using one lung SqCC patient, we screened 26 genes that are well known as potential driver cancer genes, using next-generation sequencing. Though there are many known gene panels containing more genes, we limited our analysis to the current panel, to achieve high sequencing coverage. The panel that we used has been previously validated as clinically relevant in a wide variety of solid tumors. Moreover, this multiple-gene panel focuses on actionable hotspot mutations, which have therapeutic as well prognostic values for different solid tumors, enabling a personalized medicine approach.

We performed in-depth investigation looking for SNVs and indels on the target genes. For each gene, both coding and noncoding regions were covered. We found that 83% of the retained variants are being shared by distant metastasis and primary tumor. Moreover, for each of these concordant variants, the variation frequency shows a strong concordance between the two tumor sites, with the presence of distinguished clusters, mainly containing both low- and high-frequency variants. Among the shared variants, we detected six non-silent SNVs, among which three variants at high frequency (36–99%) in the ALK gene, and at low frequency (1–6%): two in PIK3CA and one in CDKN2A. The variants CDKN2A (p.Leu65Pro) and ALK (p.Ile1461Val) were found in another patient also with lung SqCC, with the same variant frequency (data not shown). Somatic alterations of ALK gene are reported relatively rarely in squamous cell carcinoma (SqCC) (48), but, on the other hand, CDKN2A alterations are common and have been reported in recent genomic studies of lung SqCC (49). Interestingly, in one recent study, which included three patients with lung SqCC, the rate of concordance in somatic mutations between local metastasis in distinct lung lymph nodes and primary lung cancer varies from 15 to 82%, and some nodes have highly similar somatic mutation profiles (45). In another study which analyzed two lung SqCC patients, the rate of concordance in somatic mutations between distinct lung lymph nodes and primary lung cancer was identified, with 37% for one patient and 96% for the other (46). Spatial tumor heterogeneity in lung SqCC patients occurs frequently and differs significantly from one patient to another. Typically, in routine clinical practice a single biopsy is performed, to define a driver mutation and thus to suggest the optimal treatment. The occurrence of tumor heterogeneity, which was observed in the current and previous works, could make the use of a single solid biopsy ineffective. Liquid biopsy could be an actionable option to perform a serial monitoring of the tumor burden and get a comprehensive view on the genetic changes of the tumor cell diversification that could take place during distant metastasis.

In the present report, six genetic variations affecting four genes (FGFR2, FGFR3, CDKN2A, and HRAS) are shown to have a substantial discordance between the liver metastatic site and the primary tumor of the lung SqCC. Four genetic changes were gained on the genes FGFR2, FGFR3, and CDKN2A, and two genetic variants were lost on HRAS, in the metastatic tumor. Interestingly, after four cycles of treatment, a strong signal of resolution was detected at the liver metastasis through a significant sink over 60% of the tumor size. In contrast, the primary tumor showed a progression. These mixed outcomes after treatment may be related to the intra-tumor genetic heterogeneity of this lung SqCC patient. The discordant variants found in the current report have low frequency; it is possible that primary tumor cells with high-frequency genetic variants resist during the metastasis events, and a new clone harboring new low-frequency genetic variants emerges. These low-frequency variations might make metastatic tumor cells respond differently to chemotherapy treatment than the primary tumors.

Three of the discordant genetic changes have coding consequences, as follows: FGFR3 (p. Ser249Cys), CDKN2A (p. Leu16Profs^*^9), and HRAS (p. Gln61Pro). None of these non-silent mutations were found in the study of De Bruin or Um (45, 46). One explanation could be that the high sequencing-coverage depths, which were performed in this study, have resulted in identification of non-silent mutations. The advantage of performing whole-genome or exome sequencing would allow comprehensive understanding of the functional alterations found in lung cancer, but this approach could neglect rare or low-frequency variants. Sufficient base coverage of the targeted region, defined as the number of reads covering a base, is an important prerequisite for reliable variant detection from NGS data. In the present study, the cost of high sequencing coverage limited the number of assayed genes compared to the Bruin and Um studies. Nevertheless, the mean target coverage in these studies was ~100×, while in our study all the target regions are covered in average ~6,000× and at least 500× (Figure S1).

FGFR3 (fibroblast growth factor receptor 3) is a member of the fibroblast growth factor receptor family (FGFR). Alterations in the FGFR kinase family are common in lung SqCC and comprise the most frequently altered tyrosine kinase family (3, 50, 51). The mechanism of activation of FGFR3 kinase was previously established for the observed variant FGFR3 (p. Ser249Cys). The presence of the mutant cysteine amino acid instead of serine residue allows, via disulfide bound, the receptor to dimerize abnormally, resulting in ligand-independent constitutive activation in the absence of the ligand and to structural perturbation of the dimers (49). Using cell culture and tumor xenograft models, researchers described the oncogenic nature of this observed FGFR3 mutation and showed that it could drive cellular transformation (49). Cancer cell lines harboring this variant have demonstrated sensitivity to the inhibition by FGFR inhibitors and that the cell transformation can be reversed (17, 52–55). The variant CDKN2A (p. Leu16Profs^*^9) is reported in somatic pancreas carcinoma and thymoma, also as a germline variant associated with hereditary cancer-predisposing syndrome. According to several databases, this variant is located at a relatively conserved position and is predicted to have a strong effect on protein function; the variant has a damaging, disease-causing effect on protein function (56, 57). The specific variant HRAS (p. Gln61Pro) is located at a medium-conserved position, reported as both germline and somatic mutation. This variant has been reported as having a medium effect on protein function and classified as a pathogenic allele. However, in the study of functional consequences at a specific mutation hotspot on the HRAS gene, the researchers show that Q61P and Q61E variants did not show increased transforming ability relative to HRAS wild-type (wt) mouse (58). Until recently, specialists just cataloged cancers as either wt or mutant for RAS (59), to predict resistance to targeted therapies, and no treatment anti-RAS therapies exist yet in the clinic (58). In Um's study, one genetic variant of HRAS (Q61R) was found, at low frequency, in only one SqCC patient, in the primary tumor as well as in one lung lymph node, but not in the other lymph node analyzed (45).

The metastatic event in lung cancer involves dissemination of cancer cells to anatomically distant organ sites, such as the liver and brain, and their subsequent adaptation to distant tissue microenvironments. Metastasis encompasses several biological processes, which consists of a complex series of well-organized steps: tumor cells exit their primary sites of growth by local invasion and intravasation, translocate systemically, survive in the circulation, arrest, affect the distant organ by extravasation, and adapt to survive and thrive in the initially healthy microenvironments of distant tissues (43, 44). Metastasis cascades are controlled by specific classes of genes, among which researchers have described metastasis initiation, progression, and virulence genes (60, 61). The relevant variants on FGFR3 and CDKN2A genes found in the present study are probably part of these virulence genes, which potentially act to promote metastasis, provide a selective advantage in secondary sites over the primary tumor, and enable the colonization of distant organs. Our case report opens the door to expand the number of tumor pairs to be examined. The analysis of more samples will better assess the clinical impact of the detected variants. It could also reveal more non-silent variants and unravel how these variants are involved in cancer evolution mechanisms and in the clinical outcome of SqCC patients. To this respect, TracerX is a large project aiming at analyzing tumoral heterogeneity in 850 NSCLC patients from several subtypes (http://www.cruklungcentre.org/Research/TRACERx). One of the challenges faced in oncology is that the genotype does not correlate with the phenotype, for some patients. Consequently, this could result in significant inter-patient variability of drugs response, even for patients harboring the same molecular alteration. The molecular analysis of this report is restricted to SNVs and indels. For future study, it is important to expand the genetic analysis to other alterations and to assess their correlation with gene expression and protein levels.

In this work, we shed light on cell molecular changes happening between the primary and metastasis tumors to the liver of lung SqCC, by targeting, at high sequencing depth, 26 well-known cancer-related genes. Our results show in lung SqCC the following: (1) the occurrence of genetic changes between primary tumor and distant metastasis to the liver, (2) the gain of function in the FGFR3 and probably the FGFR2 genes, and (3) the loss of function of CDKN2A. It would be interesting, for a more in-depth study, to investigate the prevalence of these variants in metastatic lung SqCC, in order to reveal the exact role of these variants in tumor evolution during each metastasis step and to assess the efficacy of treatments targeting these variants in the hope of opening opportunities for new efficient therapies for this disease.

## Data Availability Statement

The datasets presented in this study can be found in online repositories. The names of the repository/repositories and accession number(s) can be found below: https://www.ncbi.nlm.nih.gov/clinvar?term=SUB7198155%5BSubmitter%20Batch%5D.

## Ethics Statement

Ethical review and approval was not required for the study on human participants in accordance with the local legislation and institutional requirements. The patients/participants provided their written informed consent to participate in this study.

## Author's Note

Our major finding, we determined the genetic changes which occur between primary tumor and distal metastasis site in Lung SqCC.

## Author Contributions

HM and AO designed and carried out the experiments. HM and RI analyzed the data. HM, VB, RI, and AO contributed to the interpretation of the results. HM wrote the paper with input from all authors. All authors discussed the results and commented on the manuscript.

## Conflict of Interest

HM and RI are cofounders of the diagnostic device company OncoDiag SAS, Paris, France. HM is also a genetic counselor at the Al-Azhar Oncology Center, Rabat, Morocco. The remaining authors declare that the research was conducted in the absence of any commercial or financial relationships that could be construed as a potential conflict of interest.
