# Supplementary figures and images for: Next-Generation Sequencing at High Sequencing Depth as a Tool to Study the Evolution of Metastasis Driven by Genetic Change Events of Lung Squamous Cell Carcinoma
Source: Front Oncol. 2020 Aug 5;10:1215. doi: 10.3389/fonc.2020.01215 (PMC7438761; doi:10.3389/fonc.2020.01215)

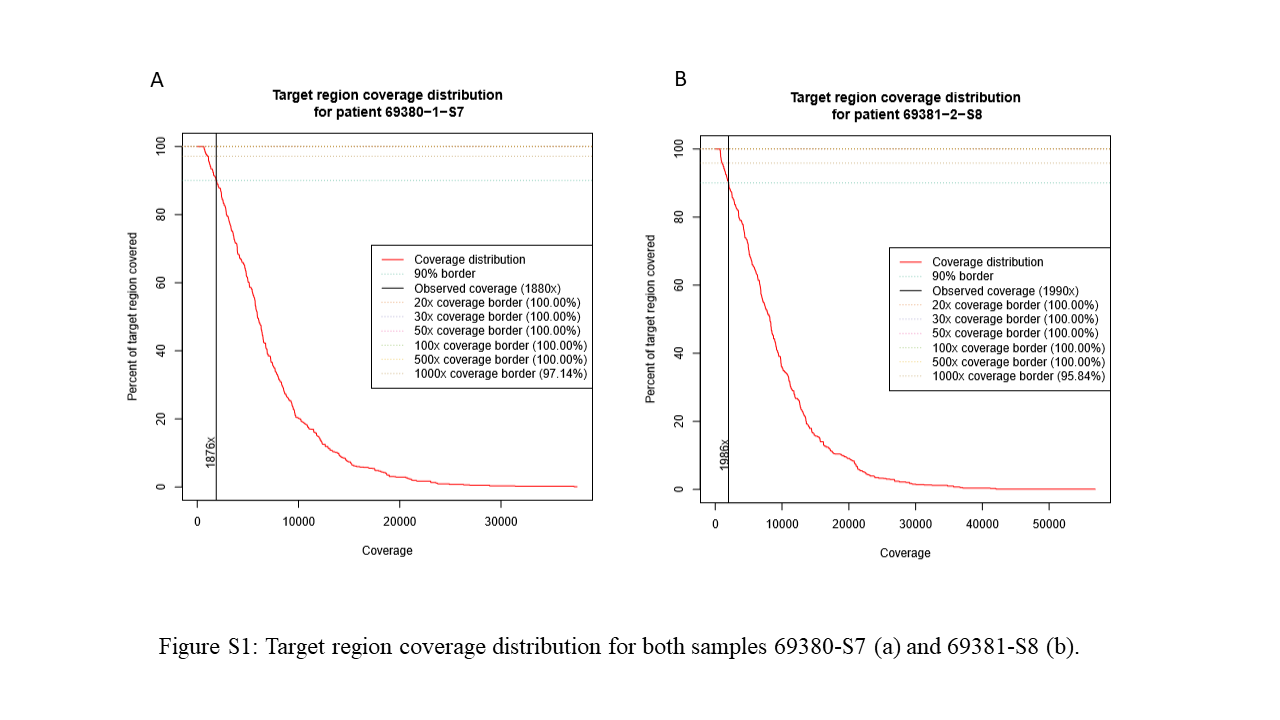

Supplement: Supplementary file 1 [file Image_1.TIF]
